# Fluctuation-response relations for a two-stage population of spiking neurons stimulated by common noise

**DOI:** 10.1007/s00422-026-01043-7

**Published:** 2026-04-28

**Authors:** Leander Dittrich, Benjamin Lindner

**Affiliations:** 1 Physics Department, Humboldt University Berlin, Newtonstr. 15, 12489 Berlin, Germany; 2 Bernstein Center for Computational Neuroscience, Berlin, Philippstr. 13, Haus 6, 10115 Berlin, Germany

**Keywords:** Stochastic spiking, Integrate-and-fire model, Fluctuation-dissipation theorem, Common noise, Neuronal signal transmission

## Abstract

Recently a method has been put forward to connect the measures of spontaneous neuronal activity and the measures of the average single-neuron response to stimuli via fluctuation-response relations (FRRs) for some integrate-and-fire (IF) type neuron models. In this work we expand this method to populations of neurons, relating their spontaneous correlation and linear-response statistics. To this end, we analyze the simple case of uncoupled cells modeled by IF neurons (first stage of processing) which receive common stochastic input and project their output spike trains onto a readout neuron (second stage of processing). We derive and verify FRRs connecting the single neuron response to cross-correlations among neurons and the response of the full system to cross-stage correlations. Furthermore, we utilize these FRRs to derive approximations of all cross-stage cross-spectra for a relevant model of a second-stage cell, the partial synchronous output (PSO). We conclude with a discussion of how our results can be expanded to more involved network settings and neuron models.

## Introduction

Stochastic neuron models have long been successful in describing various aspects of neuronal activity (Holden 1976; Tuckwell 1989; Fourcaud and Brunel 2002; Burkitt 2006; Gerstner et al. 2014). They capture certain statistics of the timing of action potentials through numerical simulations and, sometimes, analytical calculations. Especially important and accessible are (i) a neuron’s spontaneous firing characterized by the mean firing rate and the correlation function of the spike train and (ii) a neuron’s systematic response in the form of a firing-rate modulation when the cell is stimulated by a signal.

In statistical physics the response and the statistics of the spontaneous fluctuation, more specifically, the susceptibility (the Fourier transform of the response function) and the power-spectrum (the Fourier transform of the spontaneous correlation function), are connected via the fluctuation-dissipation theorem (FDT) in thermodynamic equilibrium (Landau and Lifschitz 1971), sometimes and more appropriately in some contexts referred to as a fluctuation-response relation (FRR). However, neurons operate far from thermodynamic equilibrium and, hence, the standard FDT cannot be applied to nerve cells. Also modern generalizations to some non-equilibrium situations (Agarwal 1972; Hänggi and Thomas 1982; Prost et al. 2009; Gomez-Solano et al. 2009; Engbring et al. 2023), which were experimentally confirmed by colloid systems and other many-particle systems outside of equilibrium (Mehl et al. 2010; Dinis et al. 2012), are unfortunately not suitable for spiking neurons because they are not formulated in terms of spike trains and firing rates that are the essential observables of neural systems.

Only recently, a method for deriving FRRs for IF-neuron-models has been developed and was applied to different standard types of models like the leaky IF model, the exponential IF model, models with adaptation and colored Gaussian noise (Lindner 2022) (see also for some combinations Klett and Lindner 2025), IF models with a refractory period (Puttkammer and Lindner 2024), and models with Poissonian shot noise (Stubenrauch and Lindner 2024). In most cases, the resulting relations have a similar simple form. So far, the investigation of FRRs was hence limited to single stochastic neurons.

Of course, most neurons operate in large networks together with many other units, and the last years have seen also many advances in recording the activity of many neurons in parallel, e.g. by multi-arrays (Spira and Hai 2013) or by calcium imaging (Kerr and Denk 2008). Hence, also for this situation in which we can record or model several cells at once, nontrivial relations between their spontaneous and response statistics are of interest. Furthermore, also statistics on a larger spatial scale such as local field potentials or population rates are given by functions or functionals (filtered versions) of the combined activity of many cells. Of course, also for these statistics relations between spontaneous correlation functions and the systematic response to sensory signals are of interest. In fact, coming from a different level of modeling, attempts have been made to derive fluctuation-dissipation like relations at such more coarse grained levels (Sarracino et al. 2020; Nandi et al. 2023; Deco et al. 2023; Patow et al. 2024).

Large-scale networks of neurons display vast information processing capabilities. Especially synchronous activity of neurons may encode important information and neurons in cortex may be tuned to detecting synchronous input (König et al. 1996). Thus, when investigating information processing in networks of neurons, we put special attention on synchronous activity which is accessible through population level measures.

In this work we derive a number of FRRs for a population of spiking integrate-and-fire neurons in a comparatively simple situation: the neurons are not coupled but their statistics has nevertheless a collective dimension by the fact that they are driven by a common noise. The equations derived are exact and relate cross- and power spectra of the spontaneous spike trains and voltage variables of the considered neurons to their response functions; we illustrate the validity of the FRRs by comparison to numerical simulations throughout. We also inspect a nonlinear function of the joint activity of the entire population, the partial synchronous output (PSO), which can be interpreted as a proxy for the spiking of a read-out neuron receiving the output of the population and operating as a coincidence detector (Bostner et al. 2020).

Our paper is organized as follows. The model and the statistics of interest is introduced in Sec. 2. In Sec. 3 we derive FRRs between the susceptibility and cross-spectra of single neurons within the first-stage population, while in Sec. 4 we derive a FRR between the susceptibility of the PSO (our proxy for the second-stage readout neuron) and its cross-spectra with single neurons. In Sec. 5 we find approximations for the cross-spectra of single neuron observables with the PSO that make our results more applicable. We conclude in Sec. 6 with a discussion of further extensions of the method to more involved network settings.

## Model and measures

**Fig. 1:**
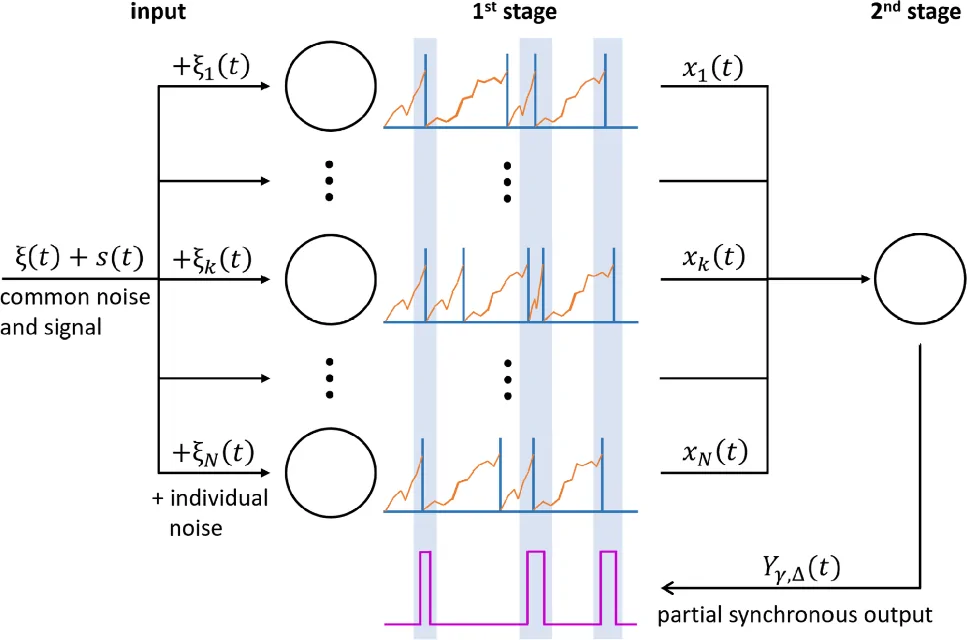
Model of information transmission through two stages Individual noise as well as common noise and a common signal (left) impinge on spiking neurons of the first-stage population (middle). The summed spike-trains are then passed on to a second-stage cell, a coincidence detector that only responds to synchronized volleys of spikes from the first population. In this work, the output of this second-stage cell is approximated by the partial synchronous output (PSO), measuring the instantaneous synchrony in the first-stage population

We consider a two-stage model of neural information processing (see Fig. 1) consisting of a first population of uncoupled spiking neurons that are subject to noise and a time-dependent stimulus and a second-stage neuron that fires only when receiving substantially synchronized activity from the first population.

For the first stage of a two-stage neural population we consider a homogeneous population of *N* uncoupled leaky integrate-and-fire (LIF) neurons. Each neuron receives individual white Gaussian noise $\xi _k(t)$ as well as white Gaussian noise ξ(t) and an input stimulus *s*(*t*) common to all neurons. To ensure that each neuron will be driven by a total noise with intensity *D*, the prefactors $\sqrt{2D(1-c)}$ and $\sqrt{2Dc}$ are introduced for the noise processes, where c∈[0,1]. Thus, the Langevin equation describing the voltage dynamics of the *k*-th neuron in the population reads

1 $$\begin{aligned} \dot{v}_k(t) = \mu - v_k(t) + \sqrt{2D(1-c)}\xi _k(t) + \sqrt{2Dc}\xi (t) + s(t). \end{aligned}$$

This is complemented by the usual fire-and-reset rule: the voltage $v_k(t)$ is reset to $v_R$ upon reaching a threshold value $v_T$. At the time of the reset, $t_{k,i}$, a spike is registered and the resulting spike train of the *k*th neuron is defined by these spike times via

2 $$\begin{aligned} x_k(t) = \sum _i\delta (t-t_{k,i}). \end{aligned}$$

For c=0 the dynamics of all neurons are completely independent, whereas for c=1 (and any non-zero noise intensity *D*) all neurons will in the long term approach identical voltage traces and spike trains even if started at different initial voltages.

For the second stage we consider a single coincidence detector cell, receiving the activity *A*(*t*) of the first-stage population as an input. The activity is given as the sum of the spike trains $x_k$ of the population filtered by some synaptic filter $\mathcal {F}$

3 $$\begin{aligned} A(t) = \frac{1}{N}\sum _{k=1}^N \mathcal {F}*x_k (t). \end{aligned}$$

Here, the asterisk ∗ denotes a convolution. The output of this coincidence detector cell *Y*[*A*; *t*] is a functional of the activity and hence a functional of all the spike trains in the first population. The exact form of this functional dependence and the synaptic filtering will only be important for approximating the cross-stage statistics. However, it will not influence the form of the resulting FRR. A possible choice for the coincidence detector neuron is a single IF neuron with strong leak and high threshold (requiring the simultaneous arrival of a critical number of presynaptic spikes to make the neuron fire). This, together with the spiking population of presynaptic cells, casts a number of mathematical difficulties. A more tractable model that approximates the IF readout neuron rather well is the above mentioned partial synchronous output (PSO) (Kruscha and Lindner 2016), defined by

4 $$\begin{aligned} Y_{\gamma ,\Delta }(t) = \Theta \left( A_\Delta (t)-\gamma +\frac{1}{2N}\right) , \end{aligned}$$

where $A_\Delta$ is the activity with a synaptic filter in the form of the box-function $\mathcal {F}(t)=\mathcal {B}_\Delta (t) = \Theta (t)\Theta (\Delta -t)$.

The PSO is active if a fraction of γ neurons in the presynaptic population fires – assuming a sufficiently small time window such that each neuron fires at most once. Such activity indicates synchrony in particular for a high threshold, $\gamma > r_0\Delta$. The spectral statistics and the information flow through the PSO is close to that of a coincidence detector IF neuron with appropriately chosen leak and threshold as was demonstrated by Bostner et al. (2020). We note that the considered simple setup (a population of uncoupled cells stimulating a coincidence detector neuron) has relevance in several sensory modalities, for instance, in the auditory spiral ganglion cells in humans (Carricondo and Romero-Gómez 2019; Kandel et al. 2000) or in the electrosensory system of weakly electric fish (Maler 2009).

We will distinguish between *spontaneous* and *driven* activity of the system. In the first case, we set the external signal to zero (s(t)=0) but still include the common noise shaping the correlations among the neurons. Although a common noise and a common signal may look similar at the first glance (in particular, when a broadband, noise-like stimulus *s*(*t*) is used), the big difference is that we assume the signal to be known and hence input-output correlations and the information transfer from signal to spike trains can be studied, whereas the common noise is unknown. In the case of spontaneous firing (s(t)=0), however, we can only calculate statistics between observables of the system (membrane voltages and spike trains for the model at hand). To characterize the spontaneous fluctuations, we will compute and measure in simulations various power- and cross-spectra of the stochastic processes involved. For two stationary stochastic processes *U*(*t*) and *V*(*t*) we calculate their power- and cross-spectra by

5a $$\begin{aligned} S_{UU}(\omega )&= \lim _{T\rightarrow \infty }\frac{\langle \tilde{U}_T(\omega )\tilde{U}_T^*(\omega )\rangle }{T},\end{aligned}$$

5b $$\begin{aligned} S_{UV}(\omega )&= \lim _{T\rightarrow \infty }\frac{\langle \tilde{U}_T(\omega )\tilde{V}_T^*(\omega )\rangle }{T}, \end{aligned}$$

where the brackets $\langle \rangle$ denote an ensemble average, the asterisk ^*^ a complex conjugation, and $\tilde{U}_T$ a finite-time Fourier-Transform given by

6 $$\begin{aligned} \tilde{U}_T(\omega ) = \int _{-T/2}^{T/2}{U(t)e^{i\omega t}dt}. \end{aligned}$$

For the white Gaussian noise processes the relations

7 $$\begin{aligned} S_{\xi _j\xi _k}(\omega ) = \delta _{jk}, S_{\xi \xi }(\omega ) = 1, S_{\xi _j\xi }(\omega ) = 0 \end{aligned}$$

will hold.

To characterize the response of the driven system to a weak stimulus *s*(*t*), we use linear response theory. The firing rate of a single neuron $r_k(t)=\left\langle x_k(t) \right\rangle$ or, more generally, any averaged observable of the system, $\left\langle Z(t) \right\rangle$ will be a functional of the injected stimulus. Assuming this functional can be expanded by a Volterra-Series, higher-order terms may be neglected and the response of any averaged system quantity will be approximated by

8 $$\begin{aligned} \langle Z\rangle [s;t] \approx \langle Z\rangle [s=0;t] +\int _{-\infty }^t{K_Z(t-t')s(t')dt'}, \end{aligned}$$

where $K_Z(t)$ is the linear-response function of the observable *Z*(*t*) with respect to a current stimulus. This equation can be simplified by Fourier transforming it,

9 $$\begin{aligned} \langle \tilde{Z}\rangle [s;\omega ] \approx \chi _{Z}(\omega )\tilde{s}(\omega ) \text { for } \omega \ne 0, \end{aligned}$$

introducing the susceptibility $\chi _{Z} = \tilde{K}_{Z,\infty }$ (the true Fourier transform of the response function over an infinite time window).

The FRRs that we aim at connect the spectra of the spontaneous statistics to the response function(s) of different observables.

## Fluctuation-response relations for the neurons of the first-stage population

We will start by applying the method for a single neuron described by Lindner (2022) to the Langevin equation of a neuron within the first-stage population in the absence of a signal (spontaneous activity with s(t)≡0). Following (Lindner 2022), we formally incorporate the voltage reset by subtracting a suitably scaled version of the spike train in the Langevin equation (1), Fourier transform the resulting equation and take its complex conjugate to obtain

10 $$\begin{aligned} (1 + i\omega ) \tilde{v}_k^* = \sqrt{2Dc}\tilde{\xi }^* + \sqrt{2D(1-c)}\tilde{\xi }_k^* - (v_T-v_R)\tilde{x}_k^*. \end{aligned}$$

Lindner (2022) multiplied the resulting equation for a single neuron with the Fourier-Transform of its own spike train. We will multiply the equation individually with the stochastic processes $\tilde{x}_j$, $\tilde{v}_j$, and $\tilde{\xi }$, and then follow the method by taking the ensemble average and divide it by *T* with $\lim _{T\rightarrow \infty }$. This leads to a system of equations of cross- and power spectra:

11a $$\begin{aligned} \begin{aligned} (1 + i\omega ) S_{x_jv_k}&= \sqrt{2Dc}S_{x_j\xi }+ \sqrt{2D(1-c)}S_{x_j\xi _k}\\ &-(v_T-v_R)S_{x_jx_k},\\ \end{aligned}\end{aligned}$$

11b $$\begin{aligned} \begin{aligned} (1 + i\omega ) S_{v_jv_k}&= \sqrt{2Dc}S_{v_j\xi }+ \sqrt{2D(1-c)}S_{v_j\xi _k}\\ &- (v_T-v_R)S_{v_jx_k},\\ \end{aligned}\end{aligned}$$

11c $$\begin{aligned} \begin{aligned} (1 - i\omega ) S_{v_k\xi }&= \sqrt{2Dc}S_{\xi \xi }+ \sqrt{2D(1-c)}S_{\xi _k\xi }\\ &- (v_T-v_R)S_{x_k\xi }.\\ \end{aligned} \end{aligned}$$

Because we assume all involved noise processes to possess Gaussian statistics, we can express the cross-spectra between those noise processes and the observables of our system by the respective response functions or susceptibilities by virtue of the Furutsu-Novikov theorem (Furutsu 1963; Novikov 1965). Specifically, for our observables the theorem entails the following relations:

12 $$\begin{aligned} \begin{aligned} S_{x_j\xi } = \sqrt{2Dc}\chi _x, \hspace{1em} S_{x_j\xi _k}&= \sqrt{2D(1-c)}\delta _{jk}\chi _x,\\ S_{v_j\xi } = \sqrt{2Dc}\chi _v, \hspace{1em} S_{v_j\xi _k}&= \sqrt{2D(1-c)}\delta _{jk}\chi _v. \end{aligned} \end{aligned}$$

Inserting the relations for the noise spectra (7) and the Furutsu-Novikov theorem (12) into the system of equations for the cross-spectra (11) leads to two FRRs and one response-response-relation (RRR):

13a $$\begin{aligned} \chi _{x}&= \frac{(v_T-v_R)S_{x_jx_k} + (1+i\omega )S_{x_jv_k}}{2D[(1-c)\delta _{jk} + c]},\end{aligned}$$

13b $$\begin{aligned} \chi _{v}&= \frac{(v_T-v_R)S_{v_jx_k} + (1+i\omega )S_{v_jv_k}}{2D[(1-c)\delta _{jk} + c]},\end{aligned}$$

13c $$\begin{aligned} \chi _{v}&= \frac{1 - (v_T-v_R)\chi _{x}}{1-i\omega }. \end{aligned}$$

We note that for j=k eq. (13a) reduces to the FRR derived by Lindner (2022) whereas eq. (13b) and eq. (13c) are the FRR for the voltage variable and the RRR between the two susceptibilities calculated recently by Klett and Lindner (2025). The above relations go beyond that by connecting for j≠k the cross-spectra of distinct neurons to the different susceptibilities.

Remarkably, the above equations can be rearranged to express the cross- and power spectra of the voltages by spike-train statistics, making voltage statistics accessible via spike-train data:

14 $$\begin{aligned} \begin{aligned} S_{v_jv_k}&= \frac{(v_T-v_R)^2}{1+\omega ^2}S_{x_jx_k}\\&- 2D\frac{(1-c)\delta _{jk} + c}{1+\omega ^2}\left[ 2(v_T-v_R)\textrm{Re}\left( \chi _{x}\right) - 1\right] \end{aligned} \end{aligned}$$

**Fig. 2:**
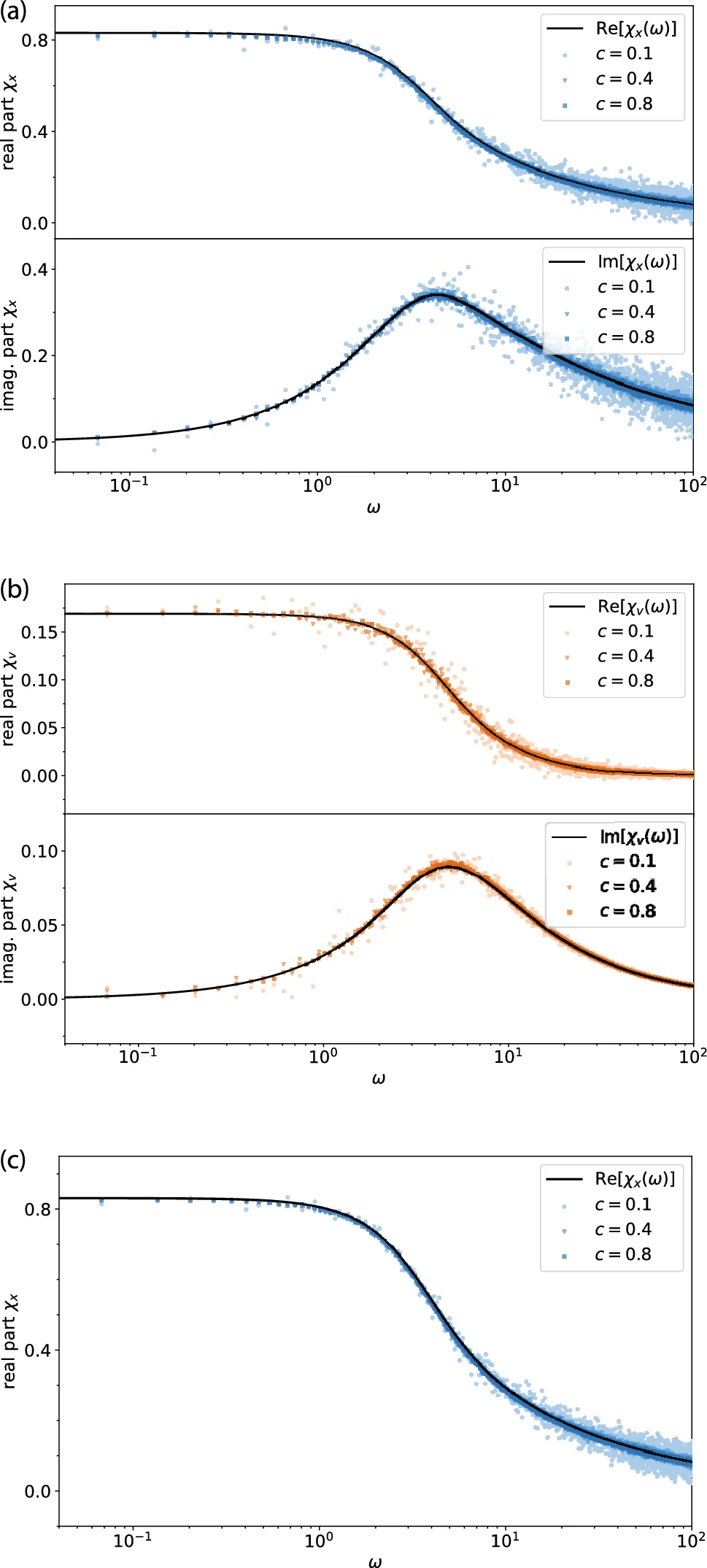
Fluctuation-response relation for the population Confirmation of the FRRs eq. (13a) (a), eq. (13b) (b) and eq. (15) (c) by comparing susceptibility (black line) to fluctuation statistics (dots) for real (top) and imaginary part (bottom) depending on frequency for multiple *c* values. Simulation for $10^5$ trials with T=100, μ=0.8, and D=0.1. The susceptibility is obtained by analytical expressions (37) and (13c)

Equivalently, eq. (14) can be formulated as another FRR connecting for j≠k the real part of the spike-train susceptibility to the spontaneous cross-spectra of spike trains or voltages of different neurons:

15 $$\begin{aligned} \textrm{Re}\left( \chi _{x}\right) = \frac{2Dc + (v_T-v_R)^2 S_{x_jx_k} - (1+\omega ^2)S_{v_jv_k}}{4Dc(v_T-v_R)}. \end{aligned}$$

Equations eq. (13a), eq. (13b), and eq. (15), are verified in Fig. 2(a), Fig. 2(b), and Fig. 2(c), respectively, for the case of j≠k by comparing the cross-spectra of the spontaneous firing in the population with the analytically known expression for the susceptibility (Lindner and Schimansky-Geier 2001; Brunel et al. 2001).

It should be noted that for the case of j≠k the analytical expressions for the cross-neuron cross-spectra are unknown. However, for c≪1 the cross-spectrum can be well approximated by (Vilela and Lindner 2009; Ostojic et al. 2009)

16 $$\begin{aligned} S_{x_jx_k}\approx 2cD|\chi _x|^2. \end{aligned}$$

A similar approximation can be used to approximate cross-spectra involving the voltage

17 $$\begin{aligned} S_{x_jv_k}\approx 2Dc\chi _x\chi _v^*, \hspace{1em} S_{v_jv_k}\approx 2Dc|\chi _v|^2. \end{aligned}$$

**Fig. 3:**
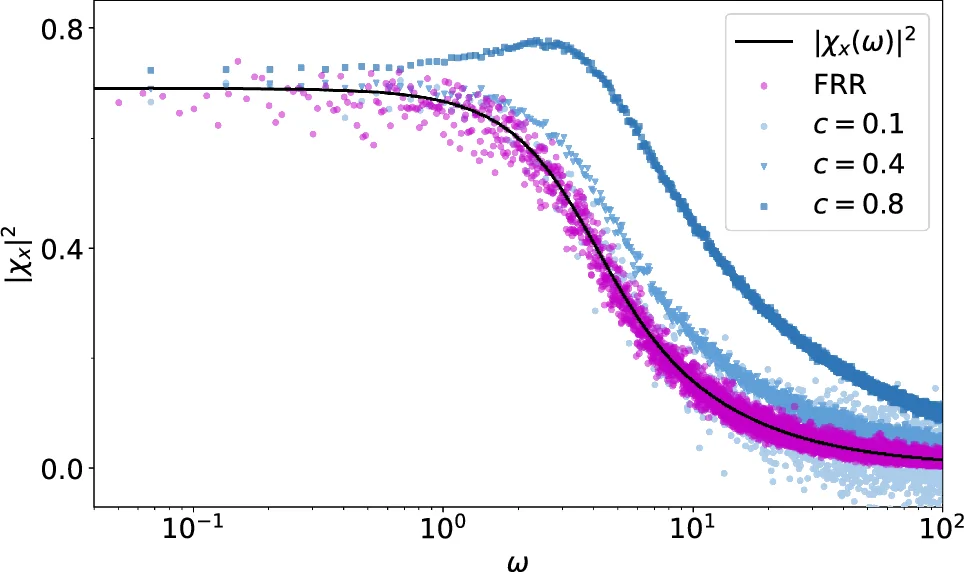
Approximate fluctuation-response relation for cross-neuron statistics The square of the absolute value of the susceptibility (black line) is approximated by rearranging eq. (16) for multiple values of c (blue). This is compared to the exact formula given by squaring the absolute value of the rhs. of eq. (13a) (purple). Simulations for eq. (13a) with T=500 and c=0.4; for all other parameters and eq. (16) parameters are chosen as in Fig. 2

We note that these approximations are consistent with the system of equations (13). However, we would like to emphasize that the latter are exact for the full range of *c* values, c∈[0,1]. The relations (16) and (17) can be regarded as approximate FRRs that trivially result from the realization-wise linear-response ansatz. The difference between eqs. (16) and (13a) is illustrated in Fig. 3. It can be seen that eq. (16) only holds for small values of *c* (light blue data points obtained from the spike-train cross-spectrum agrees with solid line, i.e., the analytical expression for the susceptibility), while (13a) holds for arbitrary values of *c* (in the figure we used c=0.4).

## Cross-stage FRR

We now turn to relations that connect observables of the two stages of information processing, namely, our proxy for the coincidence detector cell, the PSO *Y*(*t*) and the spike trains and membrane voltages of the first population. To derive such relations, we multiply eq. (10) by the Fourier-Transform of the PSO, $\tilde{Y}$, take the ensemble average, divide the equation by *T*, and take the limit $T\rightarrow \infty$ to obtain

18 $$\begin{aligned} \begin{aligned} (1 + i\omega ) S_{Yv_k}&= \sqrt{2Dc}S_{Y\xi } + \sqrt{2D(1-c)}S_{Y\xi _k}\\ &- (v_T-v_R)S_{Yx_k}. \end{aligned} \end{aligned}$$

The cross-spectra that involve the Gaussian noise processes can be rewritten by using the Furutsu-Novikov theorem

19 $$\begin{aligned} S_{Y\xi } = \sqrt{2Dc}\chi _{Y,N}, \end{aligned}$$

where $\chi _{Y,N}$ is the susceptibility of the PSO to a stimulus entering all neurons of the first-stage population. In the same way we can write

20 $$\begin{aligned} S_{Y\xi _k} = \sqrt{2D(1-c)}\chi _{Y,1}, \end{aligned}$$

where $\chi _{Y,1}$ is the susceptibility of the PSO to a stimulus applied to only one neuron of the first-stage population. These relations can be inserted into eq. (18), leading to the FRR:

21 $$\begin{aligned} c\chi _{Y,N} + (1-c)\chi _{Y,1}=\frac{(v_T-v_R)S_{Yx_k} + (1 + i\omega ) S_{Yv_k}}{2D}. \end{aligned}$$

**Fig. 4:**
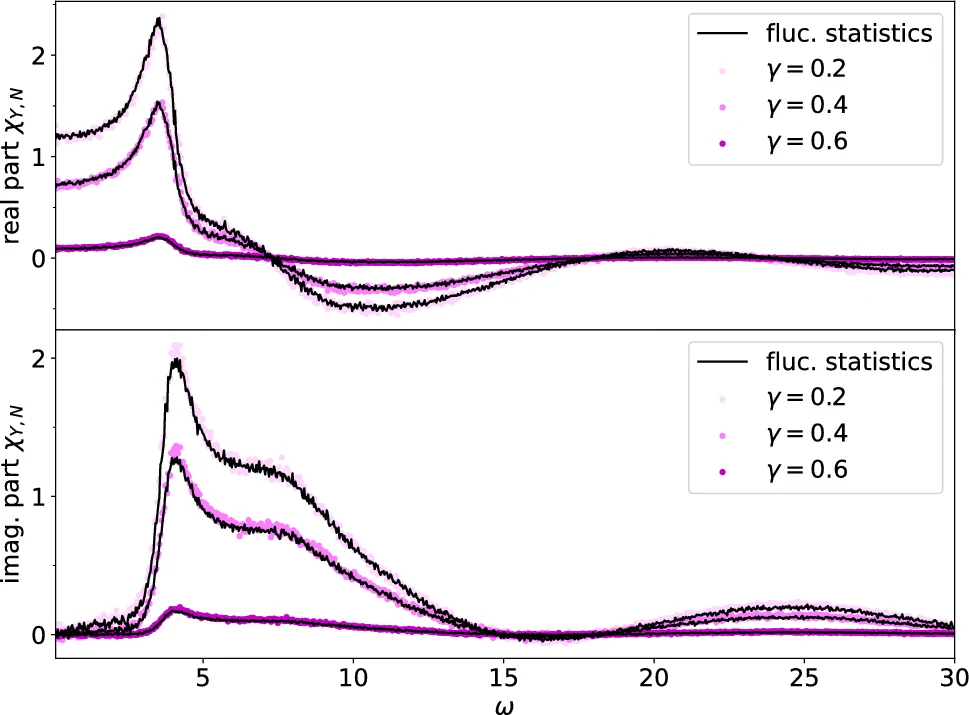
Fluctuation-response relation including cross-stage statistics Confirmation of the FRR eq. (23) by comparing susceptibility (dots) to fluctuation statistics (lines) for real and imaginary parts as functions of frequency for multiple values of γ. Mean-driven and low-noise regime (μ=1.2,D=0.01) with N=10 and c=0.1; simulations for $10^5$ trials with T=200, $\Delta t=5\cdot 10^{-4}$. The susceptibility is obtained by a broadband stimulus of uniform power for $|\omega |< 2\pi \cdot 100$ with small variance $\langle s^2(t)\rangle =0.1$

Fortunately, by the linearity of the response to a weak signal and the homogeneity of the first-stage population, the two susceptibilities are connected by

22 $$\begin{aligned} \chi _{Y,N} = N\chi _{Y,1}. \end{aligned}$$

Hence, the FRR can be simplified to include only one of the susceptibilities

23 $$\begin{aligned} \chi _{Y,N} = \frac{(v_T-v_R)S_{Yx_k} + (1 + i\omega ) S_{Yv_k}}{2D\left( c+\frac{1-c}{N} \right) }. \end{aligned}$$

We can use this equation to derive an FRR which only depends on averaged population quantities like *A* instead of the single neuron measures. When adding up the equation for all neurons, multiplying it with $\tilde{\mathcal {F}}^*$, and dividing it by *N*, the cross-spectrum $S_{Yx_k}$ turns into $S_{YA}$.The new equation then reads

24 $$\begin{aligned} \chi _{Y,N} = \frac{(v_T-v_R)S_{YA} + (1 + i\omega ) S_{YV}}{2D\left( c+\frac{1-c}{N} \right) \tilde{\mathcal {F}}^*}, \end{aligned}$$

where we have introduced the averaged filtered voltage $V=\frac{1}{N}\sum _{k=1}^N\mathcal {F}*v_k$. Equation (23) is verified in Fig. 4 by the comparison of the left and right hand sides, determined from simulations under stimulation by a broadband stimulus *s*(*t*) (measuring the susceptibility of the PSO) and in the stimulus-free spontaneous state, s(t)≡0 (measuring the spontaneous cross-spectra between PSO and neural spike trains and voltages of the first population), respectively. This comparison shows an excellent agreement and thus confirms eq. (23) for a particular set of parameters.

We note that so far we have not used any property of the PSO – the calculation and the derived relations eq. (21) and eq. (23) hold true for homogeneous functionals *Y*(*t*) of spike trains and voltages, i.e., *Y* has to depend on spike train and voltage in the same way for all neurons. In the next section we seek to simplify the FRRs using known approximations for the functionals.

## Approximating cross-stage statistics

In eq. (23) cross-spectra between first- and second-stage processes appear. However, no information about the process *Y*, other than it being a functional of the activity *A*, was used. To gain a deeper understanding of what this equation entails in the context of a firing neuron as the second-stage cell, we will approximate the behavior of such a cell by the PSO. Approximations for the susceptibility of the PSO to a signal entering all neurons and for the power-spectrum of the PSO were already derived by Kruscha and Lindner (2016). Our goal now is to derive analytical approximations for the other cross-stage spectra appearing in the FRR eq. (23) by means of this very FRR. The previous approximation of the susceptibility of the PSO relies on the fact that the activity can be approximated as a Gaussian process if the level of common noise is small (c≪1) and the number of neurons in the first-stage population is large (N≫1). Indeed, for a large number of independent filtered spike trains, central-limit-theorem arguments will apply. In this approximation the probability density of the activity is given by (Kruscha and Lindner 2015, 2016)

25 $$\begin{aligned} p_A(A) \approx \frac{1}{\sqrt{2\pi \sigma _A^2}}\exp \left( -\frac{(A-R_0)^2}{2\sigma _A^2}\right) , \end{aligned}$$

where $$\sigma _A = \frac{R_0-R_0^2}{N} + \int _{-\infty }^\infty |\tilde{\mathcal {B}}(f)\chi _x(f)|^2df$$.

Then, the PSO is just a transformation of this Gaussian process given by eq. (4). The cross-spectrum of a Gaussian process with a non-linear transformation of the same (or another) Gaussian process can be calculated via the Bussgang theorem (Bussgang 1952). Specifically, the theorem states that given two correlated Gaussian processes *U*(*t*) and *V*(*t*) with cross-spectrum $S_{UV}$, a transformed (generally non-Gaussian) process *Y*(*U*(*t*)) and the second process *V*(*t*) will have a cross-spectrum directly proportional to $S_{UV}$ with the factor of proportionality $\alpha _Y$, depending solely on the transformation

26a $$\begin{aligned} S_{Y(U),V}&= \alpha _YS_{U,V}, \end{aligned}$$

26b $$\begin{aligned} \alpha _Y&= \frac{1}{\sigma _U^2}\int _\infty ^\infty Y(U)(U-\langle U\rangle )p_U(U)dU. \end{aligned}$$

The susceptibility of the whole two-stage system $\chi _{Y,N}$ can be calculated by the Furutsu-Novikov theorem, introducing the cross-spectrum of the PSO with the common Gaussian noise ξ. This cross-spectrum is then given by the Bussgang theorem. Thus, the susceptibility of the PSO can be approximated by

27 $$\begin{aligned} \chi _{Y,N} \overset{\text {eq.~(19)}}{=} \frac{1}{\sqrt{2Dc}}S_{Y(A),\xi }\overset{\text {eq.~(26)}}{\approx }\frac{\alpha }{\sqrt{2Dc}}S_{A\xi }, \end{aligned}$$

where

28 $$\begin{aligned} \alpha _\gamma \approx p_A\left( \gamma -\frac{1}{2N}\right) = \frac{1}{\sqrt{2\pi \sigma _A^2}}\exp \left( \frac{-\beta _\gamma ^2}{2}\right) \end{aligned}$$

and $\beta _\gamma$ is a useful constant defined by

29 $$\begin{aligned} \beta _\gamma = \frac{\gamma -R_0-1/(2N)}{\sigma _A}. \end{aligned}$$

The cross-spectrum $S_{A\xi }$ is again given by the Furutsu-Novikov theorem: $S_{A\xi } = \sqrt{2Dc}\tilde{\mathcal {B}}\chi _x$. This leads to the expression for the susceptibility given by Kruscha and Lindner (2016), where it was derived using the Bussgang theorem and linear-response approximations

30 $$\begin{aligned} \chi _{Y,N}\approx \alpha \tilde{\mathcal {B}}\chi _x. \end{aligned}$$

The linear-response approximations can be circumvented by the use of the Furutsu-Novikov theorem as was done here.

For the Bussgang theorem to be valid, the (exact) Gaussianity of ξ and the (approximate) Gaussianity of the activity *A* were utilized. The latter property can also be used to obtain, by applying the Bussgang theorem once more, an approximation of the cross-spectrum between the activity *A*(*t*) and the PSO $Y = Y_{\gamma }(A(t))$

31 $$\begin{aligned} S_{YA} \approx \alpha S_{AA} = \alpha |\tilde{\mathcal {B}}|^2 \left( S_{x_1x_2} + \frac{1}{N}(S_{x_1x_1}-S_{x_1x_2})\right) . \end{aligned}$$

At the same time, due to the homogeneity of the population, the cross-spectrum of the PSO with a single spike-train will be representative of the whole population. This can be expressed by the relation

32 $$\begin{aligned} S_{YA} = \frac{1}{N}\tilde{\mathcal {B}}^*\sum _{k=1}^N S_{Yx_k}=\tilde{\mathcal {B}}^*S_{Yx_1}. \end{aligned}$$

Combining the two equations, we find an approximation of the cross-stage spectrum between the PSO and the spike-train of a neuron in the first stage population

33 $$\begin{aligned} S_{Yx_k} \approx \alpha \tilde{\mathcal {B}} \left( S_{x_1x_2} + \frac{1}{N}(S_{x_1x_1}-S_{x_1x_2})\right) . \end{aligned}$$

Since the Gaussian approximation already relies on low *c*, further approximating the cross-neuron cross-spectrum $S_{x_1x_2}$ by eq. (16) will be justified. We gain an analytical expression ($\chi _x$ and $S_{x_1x_1}$ are given by (37) and (38)) to approximate the cross-stage spectrum

34 $$\begin{aligned} S_{Yx_k} \approx \alpha \tilde{\mathcal {B}} \left( \left[ 1-\frac{1}{N}\right] 2Dc|\chi _x|^2 + \frac{1}{N}S_{x_1x_1}\right) . \end{aligned}$$

Both, the approximation for $\chi _{Y,N}$ and $S_{Yx_k}$ can be inserted into the FRR (23) which can be rearranged to yield an approximation of the cross-spectrum between the PSO and the voltage of a single neuron $v_k$

35 $$\begin{aligned} \begin{aligned} S_{Yv_k}&= 2D\left( c + \frac{1-c}{N}\right) \frac{1}{1+i\omega }\chi _{Y,N} - \frac{v_T-v_R}{1+i\omega }S_{Yx_k}\\&\approx \frac{\alpha \tilde{\mathcal {B}}}{1+i\omega }\left[ 2D\left( c + \frac{1-c}{N}\right) \chi _{x}-\right. (v_T-v_R)\\&\left. \cdot \left( S_{x_1x_2} + \frac{1}{N}(S_{x_1x_1}-S_{x_1x_2})\right) \right] . \end{aligned} \end{aligned}$$

**Fig. 5:**
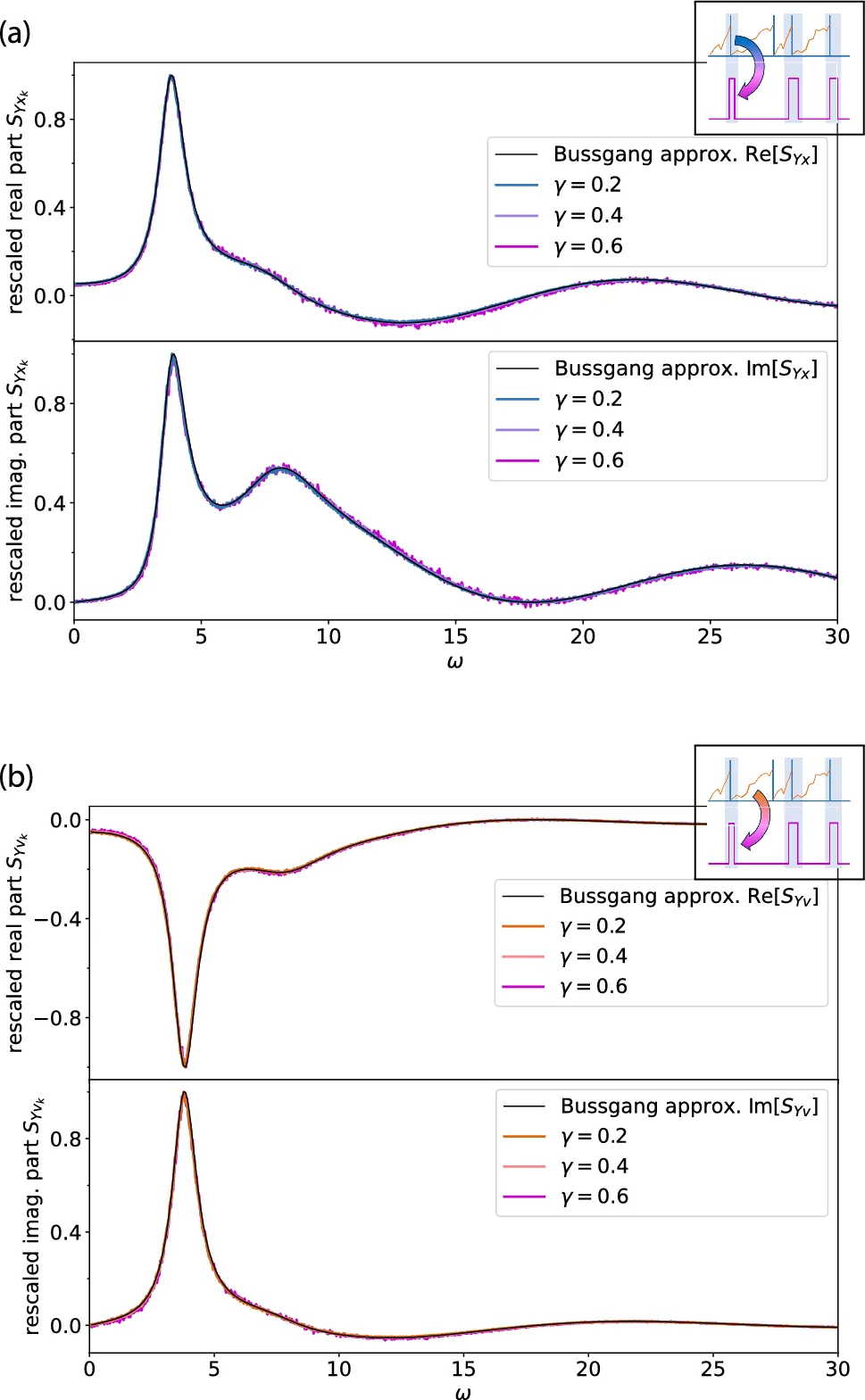
Cross-stage spectra in the Bussgang approximation Comparison of the rescaled PSO cross-spectra from simulations (colored lines) to the Bussgang approximations (black lines) for different γ values for real (top) and imaginary part (bottom) as functions of frequency. (a) $S_{Yx_k}$ by eq. (33) and (b) $S_{Yv}$ by eq. (35).

Here, we can again use eq. (16) to express the right hand side by known functions. The approximations for these cross-stage spectra by eq. (34) and eq. (35) are verified in Fig. 5(a) and Fig. 5(b), respectively, by showing the rescaled spectra for multiple values of γ and comparing with the prediction by the Bussgang approximation. Notice that the spectra only depend on γ through the linear scaling factor α, thus the rescaled spectra should lie on top of each other.

Since the spectra in Fig. 5(a) and Fig. 5(b) are rescaled by their maximum value to show the simple linear connection for all values of γ, the calculation of the scaling factor itself is not validated. In fact, this scaling factor can only be correctly approximated for $\beta _\gamma$ around zero (for γ near the mean of *A*), although the linear scaling holds regardless of γ as shown in Fig. 5(a) and Fig. 5(b). This is due to the fact that we find $\alpha (\gamma )$ to be approximated by the distribution of the activity (28) which is approximately a Gaussian. Any deviations from the Gaussian bell-shape introduced by a non-zero value of *c* will skew the distribution and will be most noticeable at the tails of the distribution, where they have the highest impact in reference to the predicted value of α. So, only for γ values close to the mean of *A* (with $\beta _\gamma < 1$) will the calculation of the scaling factor $\alpha (\gamma )$ be roughly accurate.

## Summary and conclusions

In this paper we have undertaken the first steps to generalize the theory for fluctuation-response relations developed so far exclusively to single IF neurons (Lindner 2022; Puttkammer and Lindner 2024; Stubenrauch and Lindner 2024; Klett and Lindner 2025) to the multi-neuron population level. We focused on the simple yet nontrivial case of a homogeneous population of (statistically identical) neurons that are driven by individual noise and, most important, by a common stimulus.

Instead of power and cross-spectra between spike train and membrane voltages of a single neuron, we could derive FRRs connecting in particular the response susceptibilities to cross-spectra between spike trains and/or voltages of different neurons in the population. Moreover, we found a relation that involved the spontaneous and response statistics of the PSO, interpreted as a proxy for a readout coincidence detector cell that is driven by the first-stage population. We were able to derive new approximations for all the cross-spectral statistics by using this FRR. All our relations were confirmed by numerical simulations of moderately sized populations under the influence of noise.

Our results offer alternative opportunities to determine response functions from spontaneous neural data, exploiting for instance multi-unit recordings. Furthermore, they can also be used to predict voltage cross-correlation statistics from pure spike-train statistics. Both applications concern situations in which recurrent connections are absent or negligible and the dynamics of single cells are well approximated by the leaky integrate-and-fire model.

Our model and the method to derive FRRs can be extended in several directions. Many of the computations could be repeated for neuron models that are endowed with (i) nonlinear subthreshold voltage dynamics (e.g. as in the exponential IF model Fourcaud-Trocmé et al. 2003; Badel et al. 2008; ii) spike-frequency adaptation observed in many neurons (Benda and Herz 2003; Brette and Gerstner 2005), (iii) temporally correlated (colored) noise (Hänggi and Jung 1995; Brunel and Sergi 1998; Schwalger and Schimansky-Geier 2008; Moreno-Bote and Parga 2010; Alijani and Richardson 2011; Schwalger et al. 2015), (iv) a spatial structure in the form of additional compartments coupled through gap junctions to the spike-generating compartment (Clopath et al. 2007; Ostojic et al. 2015; Doose et al. 2016). As long as the involved noise is Gaussian and we have an explicit reset rule in place, we can still invoke the Furutsu-Novikov theorem to introduce the spike-train response functions into the relations; for Poissonian shot noise and similar stochastic driving, we may use the equivalent cross-correlation–response relation, recently derived by Stubenrauch and Lindner (2024) (although these involve different response functions, namely, those with respect to a modulation of the input rate of the driving point process). With the common noise still being the main cause of nontrivial relations in this case, the mathematical structure of the FRRs is expected to be similar to what was obtained in our paper.

Beyond the integrate-and-fire framework, i.e., for populations of nonlinear multidimensional dynamical neuron model (e.g. a conductance-based Hodgkin-Huxley-type model) driven by Gaussian current noise, the application of our method could yield relations between the membrane-voltage response function and certain cross-correlation functions of the membrane potential and nonlinear functions of the membrane potential possibly combined with the gating variables. The latter cross-correlation functions characterize the spontaneous activity of the model but will be difficult to measure in experiments, especially, when gating variables are involved. Hence, the resulting FRRs for such conductance-based multidimensional models may be tested only in theory or simulations (where we know the time series of all variables including the gating variables) but not in experiments, which obviously limits their use. An alternative would be to approximate the multi-dimensional neural dynamics of such models by a multi-dimensional IF model and then to derive (approximate) FRRs, similar to the ones that were discussed here and in Lindner (2022) and that require only the knowledge of the membrane potentials and the spike trains of the neurons in the population, i.e. experimentally accessible observables.

A qualitatively new step will be to consider recurrent connections among neurons and also relax the strong assumption of population homogeneity, i.e., permit that neurons differ in their cell types (most basic, excitatory and inhibitory cells) and their parameters (mimicking variability in the respective neural subpopulations). The methods developed in our paper will be also applicable in such a setup, only that the specific relations will depend on the indices through the specific cellular parameters and connection structure. In this setting, an established theory of the cross-spectra of IF neurons exist (see Trousdale et al. 2012; Ocker et al. 2017) but this is based on a realization-wise linear-response ansatz (proposed by Lindner et al. 2005b) which can drastically fail in some situations (Lindner et al. 2005a). The key result of this theory relates the covariance matrix of spike-train cross-spectra to the matrix of spontaneous power spectra through the matrix of the network’s response functions. This relation can already be regarded as a kind of FRR, although an approximate one that somewhat trivially results from the linearity of the approximation. It will be exciting to compare the exact FRRs resulting from our method to the approximate ones of the Trousdale theory. Our exact FRRs might turn out to be useful for finding new approximations for the appearing statistics and, more generally, for better understanding the constraints of the information flow in recurrent networks. Moreover, how to exploit the FRRs in recurrent networks to infer network connectivity or individual parameters is a completely open but exciting problem for future research.

## Appendix: Firing statistics of the LIF neuron with white Gaussian noise

Numerous functions and measures of the LIF neuron driven by white Gaussian noise are known analytically. Its firing rate is given by (Ricciardi 1977)

36 $$\begin{aligned} r_0 = \left[ \sqrt{\pi }\int _{z_T/\sqrt{2}}^{z_R/\sqrt{2}}dz\, e^{z^2} \text {erf}(z)\right] ^{-1} \end{aligned}$$

with $$z_{T/R} = (\mu -v_{T/R})/\sqrt{D}$$.

The susceptibility of a LIF neuron driven by white Gaussian noise can be expressed by parabolic cylinder functions $\mathcal {D}_{\alpha }(z)$ (Whittaker and Watson 1990). This expression is given by (Lindner and Schimansky-Geier 2001)

37 $$\begin{aligned} \chi _x(\omega ) = \frac{ir_0\omega /\sqrt{D}}{i\omega -1}\frac{\mathcal {D}_{i\omega -1}(z_T) - e^{\frac{z_R^2-z_T^2}{4}}\mathcal {D}_{i\omega -1}(z_R)}{\mathcal {D}_{i\omega }(z_T) - e^{\frac{z_R^2-z_T^2}{4}}\mathcal {D}_{i\omega }(z_R)}. \end{aligned}$$

Alternatively an expression via confluent hypergeometric functions exists (Brunel et al. 2001). The power-spectrum of such a neuron is given by (Lindner et al. 2002)

38 $$\begin{aligned} S_{xx}(\omega ) = r_0\frac{|\mathcal {D}_{i\omega }(z_T)|^2 - e^{\frac{z_R^2-z_T^2}{2}}|\mathcal {D}_{i\omega }(z_R)|^2}{|\mathcal {D}_{i\omega }(z_T) - e^{\frac{z_R^2-z_T^2}{4}}\mathcal {D}_{i\omega }(z_R)|^2}. \end{aligned}$$

## Data Availability

All data was generated by simulations and is displayed in the figures. The code will be provided upon request.
